# Sex Hygiene Endorsed

**Published:** 1914-07

**Authors:** 


### Sex Hygiene Endorsed.

The sex hygiene question has reached national and international proportions. An earnest discussion is being waged in every quarter as to when, how, and where this instruction should be given. And, as would be expected, quite a ripple of opposition is met with here and there. The most significant action taken recently is the serious way in which the National Bureau of Education has taken up the matter. We have long needed just such support, for it is only by painstaking collection of data on any subject that a valid conclusion can be based.

The National Teachers' Association, through its department of patrons, endorsed the movement of sex education, and is endeavoring to solve the problem as how best to incorporate such instruction in the curricula of our schools.

The National Federation of Women's Clubs, during its recent congress in Chicago, endorsed this movement and reported some very substantial work done in collecting information and in having established the teaching of sex hygiene in the normal schools.

The National Conference of Charities and Correction has considered more specifically the suppression of vice. It declares that there *is* an urgent "social emergency," and is proceeding in a business-like way to bring about substantial reform.

The International Sunday School Association takes the position that sex knowledge should be taught in the Sunday school for the reason that it should be taught *right,* that it may lead to purity and righteousness. The principal report at its recent conference, in part, very tersely says: "With the new awakening and discussion of sex matters the pendulum has swung from silence to a publicity that it almost nauseating. Literature, the stage, the newspaper, the 'movies' have exploited the interest in the subject in the endeavor to avoid false modesty, and may in the end break down the barriers of real modesty.

"With the religious atmosphere and reverent, receptive attitude of the Sunday school, it is eminently fitted to bear the message of the knowledge that tends to personal purity. It is the plainest religious strategy."

